# The Prevalence of Corneal Disorders in Pugs Attending Primary Care Veterinary Practices in Australia

**DOI:** 10.3390/ani15040531

**Published:** 2025-02-13

**Authors:** Wai In Lau, Rosanne M. Taylor

**Affiliations:** Sydney School of Veterinary Science, The University of Sydney, Sydney, NSW 2006, Australia

**Keywords:** VetCompass, ophthalmology, brachycephalic, keratitis, corneal pigmentation

## Abstract

Pugs are highly predisposed to corneal disorders, such as brachycephalic ocular syndrome (BOS), due to their disproportionate skull, reduced corneal sensitivity and eyelid anomalies such as distichiasis, entropion and lagophthalmos. The risk of corneal disorders which cause significant suffering is substantial, prompting calls for international efforts to reduce their prevalence. While these debilitating conditions are also likely to be common in pugs in Australia, their prevalence and risk factors have not been reported. The anonymised electronic patient records (EPRs) of 1318 pugs attending 139 primary care veterinary clinics participating in VetCompass Australia (VCA) in 2017 were used to investigate the prevalence of ophthalmological pathologies and associated demographic risk factors. Pugs were diagnosed with overweight/obesity (prevalence: 20.2%, 95% confidence interval {CI}: 18.1–22.4) ophthalmological abnormalities (14.5%, 95% CI: 12.6–16.3), particularly corneal disorders (12.4%, 95% CI: 12–15.7), and other conditions. The most prevalent ophthalmological disorders were corneal ulcers (5.5%, 95% CI: 4.4–6.9), corneal pigmentation (3.6%, 95% CI: 2.8–4.8) and keratoconjunctivitis sicca (KCS) (3.3%, 95% CI: 2.5–4.5). The risk of all corneal disorders increased with age (odds ratio 1.11, 95% CI: 1.07–1.15), and corneal pigmentation, KCS and keratitis were more prevalent in older pugs (a median age of 7.6 years or older), while entropion, neovascularisation and ulcerative keratitis affected younger dogs (a median age of 3 years or under). The underlying BOS conformation defects, exophthalmos, lagophthalmos and distichiasis, and early signs of corneal damage should be identified through detailed examinations during primary care veterinary visits, and affected pugs should be removed from the breeding population and treated to reduce the severity and duration of their suffering.

## Simple Summary

Pugs are increasingly popular as companions due to their baby-like features such as their protruding round eyes and flat face. However, these attractive features contribute to various respiratory, skin, gastrointestinal, neurological and eye disorders in this breed. Pugs are at risk of superficial eye disorders due to their bulging eyes, reduced corneal sensitivity and abnormal eyelid closure. This study investigated the prevalence and risk factors for ocular conditions in pugs. The primary veterinary care records of pugs attending primary care veterinary clinics participating in VetCompass Australia in 2017 were analysed. There was a high prevalence of overweight/obese pugs (20.2%) and eye and eyelid abnormalities (14.5%). The most prevalent ocular disorders were corneal ulcers, corneal pigmentation and keratoconjunctivitis sicca (dry eyes). The risk of the most prevalent corneal disorders increased with age, including corneal pigmentation, dry eyes and keratitis (inflammation of the cornea). Many other eye conditions related to pug facial conformation were identified in veterinary visits that contribute to chronic suffering and poor welfare. Pug health can be improved by early, more detailed veterinary monitoring of eye abnormalities, enabling earlier treatment and the prompt removal of pugs with eye abnormalities from the breeding population.

## 1. Introduction

The rising popularity of brachycephalic dog breeds as companions is in part due to their paedomorphic features including a rounded wide skull, shortened rostrum and protruding eyes [1,2,3]. Pugs have become one of the most popular brachycephalic breeds in the last decade; they were one of the top three toy breeds registered in the past five years in the UK [4] and one of the top five most popular toy breeds registered in Australia [5]. Pugs are predisposed to more than 25 disorders in the broad groups of respiratory, neurological, ophthalmological, gastrointestinal and dermatological problems [2,3,6,7], with many problems related to their brachycephalic morphology. Pugs are particularly at risk of brachycephalic obstructive airway syndrome (BOAS) and corneal disorders even among brachycephalic breeds [3,8,9] with international calls for action to reduce the suffering caused by their prevalence. The three most prevalent disorders in pugs in the UK in 2013 were overweight/obesity, corneal disorder and otitis externa [8]; however, while these conditions have been identified to be common in the breed, their prevalence has not been reported in Australia.

The disproportionate skull of brachycephalic breeds typically has shallow orbits, which results in the bulging of the globes (exophthalmos) and exotropia (lateral strabismus) [1,2,10]. Consequently, the eyes are exposed to repeated external insults resulting in corneal infection and ulceration (ulcerative keratitis) [10]. This prominent eye conformation and macroblepharon (euryblepharon or abnormally large opening of eyelids) also contribute to lagophthalmos (the inability to close the eyelids completely), which compromises the spread of the protective tear film, resulting in regions of corneal drying followed by secondary erosion and ulceration [11]. Additionally, the excessive skin folds around the muzzle (caused by its compression), causes hairs to rub against the cornea (trichiasis), particularly hairs of the nasal skin folds [1,2]. Long-term trichiasis can result in corneal scarring, perforation, pigmentation or even blindness [1,10].

Common corneal disorders linked to brachycephalic eye conformation in pugs with excessive exposure of the sclera and cornea [12] are described as brachycephalic ocular syndrome (BOS). These include bacterial keratitis, conjunctivitis, limbic epithelial thinning, corneal pigmentation and keratoconjunctivitis sicca (KCS) [1,10,13]. KCS disrupts the normal corneal defence mechanisms provided by the preocular tear film and increases the risk of corneal ulcers and dry eye disease (with signs of mucopurulent discharge, chemosis and keratitis). The tear film contains antimicrobial substances for immune defence and removes foreign matter from the cornea, with its lubrication propelled by effective eyelid blinking [14,15]. Damage to the cornea initially causes intense, persistent pain and discomfort as the cornea has a high density of nociceptive afferent axons [2,10,16] in most breeds; however, innervation is reduced in brachycephalic breeds. Blepharospasm, increased lacrimation, tear staining (epiphora) and photophobia are common clinical presentations of ocular discomfort and irritation [14,15,17]; however, the reduced corneal sensitivity in pugs further reduces the protective benefits of these responses. Eyelid anomalies, including distichiasis, ectopic cilia and entropion, are additional risk factors associated with keratopathy [1,10,13,18,19,20] that are common in this breed. While conformational abnormalities can be detected early, corneal pigmentation is often one of the first clinical presentations; it worsens with age, reduces patient well-being and can obscure vision [17,18,21]. It is frequently associated with progressive inflammatory corneal pathology, including chronic superficial keratitis (pannus), chronic ulcerative or nonulcerative keratitis and KCS [10,15,18,19].

A thorough and regular ophthalmological exam in primary care veterinary practice provides initial screening for ophthalmological conditions, including developmental anomalies such as persistent pupillary membranes, and this allows early stages of the disorders to be identified and managed. Many ocular disorders that pugs are predisposed to can be diagnosed with basic ophthalmological equipment that is readily available in primary veterinary practice, such as the Schirmer tear test (STT) for KCS and fluorescein staining for corneal ulcers [14,15,17]. Pigmentation of the sclera and cornea was reported in almost all pugs undergoing a direct ophthalmoscopic examination in a veterinary teaching hospital. Congenital abnormalities commonly detected in this study included persistent pupillary membranes (85.3% of the pugs) and iris hypoplasia (72.1% of the pugs) [18]. More advanced ophthalmological diagnostic tools such as slit-lamp biomicroscopy as well as corneal thickness and corneal touch threshold measurement assist in the further categorisation of the disease [18]. For instance, a meibometer can be utilised to diagnose qualitative KCS through tests for the adequacy of the lipid component of the preocular tear film [13,22,23], the tear film breakup time (TFBUT) can be used to evaluate tear film quality and corneal thickness can be measured, but these are not often reported in primary practice.

The VetCompass studies in the UK and Australia provide information on the common disorders that are recognised in dogs attending primary veterinary practice. Veterinarians record the breed of the dog based on phenotype and owner history, and in the UK, Kennel Club registration information if it is available. Ophthalmological disorders were found to be the second most prevalent disorders in pugs in a retrospective study of primary veterinary care records from VetCompass UK [8], specifically corneal disorder, ocular discharge, conjunctivitis and KCS with a prevalence of 8.7%, 2.2%, 1.9%, and 1.9%, respectively; however, the spectrum of other BOS-related corneal conditions and the association between demographics and disease prevalence were not explored. Age and sex have been reported to be associated with KCS and pigmentary keratitis [18,19,21,24,25]. Other studies of the prevalence of health disorders in brachycephalic dogs have investigated pet insurance databases and breed surveys [2,26] of pugs that were insured and registered [27,28] (usually younger populations) and described corneal disorders to be common in the breed.

This study analysed veterinary clinical data from VetCompass Australia (VCA), an animal health surveillance programme which collates data (de-identified EPR) from a large group of participating primary care veterinary practices across Australia [29,30]. We investigated the prevalence of ophthalmological and corneal pathologies and the demographic risk factors associated with corneal disorders in pugs under primary veterinary care in Australia in 2017.

## 2. Materials and Methods

### 2.1. Study Design

A retrospective study design was used to estimate the prevalence and risk factors for the diagnosis of ophthalmological disorders in pugs based on analysis of their EPR. VCA collects anonymised EPRs from primary care veterinary practices in Australia for epidemiological research on breed-specific disorders in companion animals [30,31]. The information collected in each EPR included the demographics of pugs in Australia (species, breed, sex, neuter status, date of birth (DOB), bodyweight and colour) and the clinical information (free-form text from veterinary examination notes). A total of 15754 EPRs from all 2134 pugs that had attended the 139 participating primary care veterinary clinics in Australia between 1 January and 31 December 2017 were extracted from VCA for this study. Dogs that attended more than one veterinary business in 2017 were removed if their name, DOB, sex and colour were identical but may not always have been identified due to anonymity. Dogs with at least one EPR in 2017 or another recorded client interaction, such as the sale of medication, were included in the total number of extracted EPRs.

### 2.2. Ethical Approval

Ethics approval for the VetCompass project, including this study of anonymised EPR, was granted by University of Sydney Human Ethics Committee, New South Wales, Australia (HREC protocol number 2013/919 approved from 13 November 2013–13 November 2025).

### 2.3. Sample Size Calculation

Assuming the expected prevalence of corneal disorders was 8.72% based on the VetCompass study on pugs in the UK [8], the study required was a sample size of 1273 for estimation of the prevalence of corneal disorders with 1.55% precision and 95% confidence [32]. A total of 1320 patients were randomly selected, drawn from primary care clinics in New South Wales, Queensland and Victoria, and 2 were excluded due to duplicated selection. Pugs were randomly selected for inclusion using the Excel RAND and filter function (Microsoft Office Excel 2016 version 16.51, Microsoft Corp, Redmond, WA, USA).

### 2.4. Data Collection and VeNom Coding

The veterinary examination notes from each EPR in 2017 for the randomly selected dogs were assessed in Excel. VeNom codes were assigned to each patient for 2017 based on the examination notes to classify the reason for visit, presenting complaints, procedures, diagnostic tests and diagnoses [33], with a focus on the diagnosis of ophthalmological conditions. To prevent counting ongoing cases more than once, continued treatment for the same diagnosis or recurring diagnosis for the same patient was only counted once in the year of study for an individual. Pre-existing and new clinical disorders were not differentiated.

Specific eye conditions were categorised based on the parts of the eye affected as follows: orbit and other, eyelids, conjunctiva and nictitating membrane, tear and nasolacrimal systems, cornea, sclera, lens, uvea and retina, and information on the pathologies was tabulated. A corneal disorder was recorded when conditions involving the cornea were diagnosed in the patient notes. Disorders included keratopathy, keratitis, corneal pigmentation, keratoconjunctivitis, ulcerative keratitis, corneal scarring and corneal lipidosis. Keratitis included pannus, pigmentary and unspecified keratitis. The code for KCS was given to a patient when a diagnosis was given, tears were produced at less than 15 mm/minute using the Schirmer tear test (STT) or dogs were treated with eye drops containing cyclosporine or tacrolimus [14,15]. Cases of corneal ulcers were identified based on the diagnoses reported as well as a positive fluorescein test reported in the examination notes [17]. ‘Blepharospasm’ was coded when ‘squinting’ or ‘excessive blinking’ was mentioned. ‘Episcleritis’ was coded when ‘inflamed sclera’ was recorded. Dogs with ‘bulging eyes’ were coded as having ‘exophthalmos’. Dogs with ‘lens degeneration’ were coded as having ‘nuclear sclerosis’.

The median age of dogs with the ten most prevalent ophthalmological disorders was calculated in years using DOB from EPRs. Difficult-to-categorise cases were reviewed by a third-party veterinarian before their inclusion.

The patient coat colour was grouped to the breed standard colours of the Australian National Kennel Club [34] and other non-standard colours using the following categories: silver, apricot, fawn, black, brindle, brown and others. Age was calculated using the DOB and latest visit dates of each dog. Age was analysed as both a continuous and categorical variable. Age was further categorised into three life stages: puppy (1–12 months), adult (1–6 years old) and senior (7+ years old) [35]. Neuter status described the status of the dog (entire or neutered) at the final EPR, with the age at neutering unavailable (unless performed in 2017).

The bodyweight of the patients last recorded in 2017 was used. Dogs under 12 months old were excluded for analysis of bodyweight as a risk factor for disease. Bodyweights were not used in identifying overweight dogs due to the considerable range beyond the breed standard weight in the population. Overweight and obese patients were determined using the following criteria: dogs diagnosed as overweight/obese by a veterinarian, dogs whose records ‘recommend weight loss’ or ‘weight control’, dogs that were on veterinary-practice-provided weight loss diet and dogs that had a body condition score (BCS) of 4/5 or 6–7/9 for overweight and 5/5 or 8–9/9 for obesity [36]. The body condition of the patients was grouped into 4 categories: neither overweight or obese, overweight, obese and not reported.

### 2.5. Statistical Analysis

Study data were then transferred to RStudio Version 1.4.1717 for statistical analyses. Demographic variables were described for the sample group, including the sex, neuter status, age, body condition and coat colour. Prevalence values with 95% confidence intervals (CIs) and the median age were tabulated for the ten most prevalent ophthalmological disorders. A chi-square test compared the prevalence values of corneal disorders between the population of pugs in Australia and the UK [8] using Statulator [37]. Statistical significance was set at the level of 0.05. Complete data were available for sex, neuter status and age for all dogs. Dogs with missing data were not included for analysis of body condition (941 missing) and coat colour (10 missing). Binary logistic regression modelling was used to evaluate the univariable association between risk factors (age, sex, neuter status, body condition and coat colour) and the diagnosis of corneal disorders. The rare and non-standard coat colours (apricot, brindle and silver) were incorporated into “other” category for coat colour while obesity was grouped into the overweight category for body condition. Risk factors with liberal associations (*p* < 0.2) were taken from univariable modelling for inclusion in multivariable evaluation to determine if there was a significant association between variables (*p* < 0.05). Interactions between final variables were identified using manual backward stepwise elimination.

## 3. Results

In total, 11737 EPRs from 1318 pugs were included in this study, comprising 59.7% of all pugs attending 139 participating VetCompass practices from three Australian states in 2017. The number of pugs registered with Dogs Australia in 2017 was 1506, 10.9% higher than the average of the previous 5 years. The percentages of female and male dogs that were neutered were 73.7% (*n* = 448) and 73.8% (*n* = 524), respectively, higher rates than reported in UK pugs (neutered males: 30.7% and neutered females: 28.6%) [38].

### 3.1. Demography

Over 50% of pugs were adults (*n* = 661), 26.5% were seniors (*n* = 349) and 23.3% were puppies (*n* = 308). The median age of the pugs was 3 years (IQR = 6, range = 0–17), the same as a UK VetCompass study of pugs in 2013 [8]. Coat colour data were available for 99.2% of the pugs, with fawn being the most common colour (*n* = 796, 60.4%) followed by dark colours (black and brown: 36%), while silver and apricot were rare (2.1%), which are similar findings to the UK VetCompass pugs (63.1% fawn and 27% black; other colours rare). The median bodyweights of pugs in adult males and adult females are 10 kg and 8.6 kg, respectively, which are higher than those in the reported data in the UK VetCompass pugs (median bodyweight of 9.9 kg in adult males and 9.2 kg in adult females). The proportion of pugs that were identified as overweight, including those that were obese, was 20.2% (*n* = 266, 95% CI: 18.1–22.4). The overweight pugs were more often male (*n* = 150, 56%), and most were adults (60.5%). There was a higher proportion of overweight adults (24.4%) and seniors (23.3%), while puppies were less frequently reported as overweight (7.8%). Body condition data were available for 377 dogs (28.6%) with 70.6% of these dogs being reported by the veterinarian to be overweight (*n* = 266) and 10.6% being obese (*n* = 40). The body condition of 71.4% of the dogs was not reported. In pugs older than 12 months old (*n* = 1010) with a bodyweight 15% or above the breed standard of 6–8 kg (9.3 kg or above), 4% had a normal BCS, 86% did not have their BCS recorded and the rest were reported to have an overweight BCS.

### 3.2. Prevalence of Ophthalmological Disorders

The highest prevalence of ophthalmological disorders was reported in the following categories: senior dogs (25.2%), obese dogs (22.5%), females (17.3%), silver dogs (28.6%) and neutered dogs (15.3%) (Table 1).

### 3.3. Common Ophthalmological Disorders

The prevalence of ophthalmological disorders in pugs attending primary veterinary clinics in Australia in 2017 was 14.5% (*n* = 191, 95% CI 12.6–16.3), and 88.6% had corneal involvement. The cornea was the most common site for ophthalmological conditions in Australian pugs compared to other compartments of the eye, with a corneal disorder prevalence of 12.4% (*n* = 164, 95% CI: 12.0–15.7). The dogs had a median of two ophthalmological abnormalities in 2017 (IQR = 1, range = 1–7).

The proportion of pugs diagnosed with corneal conditions in Australia was significantly higher (χ2 = 8.2, *p* < 0.05) than in the UK VetCompass study (8.77%). The pugs in Australia were 1.49 times more likely to have corneal disorders, compared to the pugs in the UK (odds ratio = 1.49, 95% CI: 1.13–1.95).

The most prevalent ophthalmological condition was ulcerative keratitis (*n* = 73, prevalence = 5.5%, 95% CI: 4.4–6.9) (Table 2). Four of the ten most prevalent ophthalmological disorders involved the cornea (ulcerative keratitis, corneal pigmentation, keratitis and corneal scarring), and three of the ten most prevalent clinical signs were related to ocular irritation (blepharospasm, epiphora and chemosis). The proportion of pugs reported to have eyelid-related anomalies including entropion, exophthalmos and distichiasis were 1.1%, 0.9% and 0.3%, respectively (Table 3). Among the pugs with corneal ulcers (*n* = 73), 5.5% of them also had KCS (*n* = 4). In the dogs with corneal pigmentation (*n* = 48), 18.8% of them had concurrent corneal ulcers (*n* = 9), 39.6% had concurrent KCS (*n* = 19) and 10.4% had concurrent keratitis (*n* = 5). There were three pugs with corneal ulcers that had concurrent KCS and corneal pigmentation.

Among the ten most prevalent ophthalmological conditions, five were more common in senior dogs (>7 years), which include corneal pigmentation (median age: 7.6 years), KCS (10 years), keratitis (7.7 years), nuclear sclerosis (10 years) and blindness (11.2 years) (Table 2). Three of the common ophthalmological disorders in seniors involved the cornea (corneal pigmentation, KCS and keratitis). Neovascularisation and exophthalmos were reported in dogs with a median age of 1 year. No cases of persistent pupillary membranes or iris adhesions were reported. Blindness was the tenth most prevalent ophthalmological condition.

### 3.4. Risk Factors Associated with Corneal Disorders

A univariate logistic linear regression identified significant associations of the following variables with corneal disorders: neuter status, age (categories) and age (continuous) (significant findings highlighted in bold in Table 4). A further analysis using a multivariate logistic regression on age (categories) and neuter status did not identify biologically significant interactions between these variables (Table 5). Age was found to be the only risk factor contributing to the prevalence of corneal disorders.

## 4. Discussion

This breed-specific health study in a large group of pugs in Australia identified a high prevalence of corneal disorders consistent with brachycephalic ocular syndrome that were recorded in primary care veterinary consultations. The risk of corneal disorders, as a group, increased with age, and corneal pigmentation, KCS and keratitis were more prevalent in older pugs (a median age of 7.6 years or older), while entropion, neovascularisation and ulcerative keratitis affected younger dogs (a median age of 3 years or under). Overweight and obese body conditions were common in the breed in Australia but were not a risk factor for corneal disorders. Sex, neuter status and coat colour did not modify the corneal disorder risk in the Australian pugs. Our findings of the high prevalence of corneal disorders and age as a risk factor are consistent with recent reports of corneal disease in pugs in the UK and the USA [8,24,25,26] and confirm the widespread, age-related impact of these conformation-related conditions in this breed, with implications for pug welfare. These findings provide detailed information on the most frequently identified findings of BOS during primary care veterinary examinations of this popular breed, and we identify opportunities to strengthen the early detection and treatment of these conditions.

### 4.1. Ophthalmological Conditions and Corneal Disorders

Our findings that 14.5% of pugs attending primary veterinary practices in Australia in 2017 were reported to have at least one ophthalmological condition, with 88.6% of these dogs having corneal involvement, demonstrated a higher prevalence of corneal disorders in pugs than in the UK [8] based on a similar analysis of EPRs. Pugs have been reported to be at an increased risk of corneal ulceration, ocular discharge and conjunctivitis relative to non-pugs [38]. Our study reports the prevalence of many other eye conditions that were identified in primary veterinary care visits and contribute to the poor welfare of pugs with BOS [12].

A cross-sectional study of UK VetCompass data from 2016 reported a 15.8% occurrence of ophthalmological conditions, a 6.8% occurrence of corneal ulcers and an increased odds ratio of 13.01 for corneal ulcers in pugs compared to non-pugs [38]. However, in a list of the top 30 ophthalmological conditions reported in the UK VetCompass study of pugs, the only disorders included were corneal ulceration (6.8%), conjunctivitis (2.9%) and ocular discharge (1.9%). Many of the other common features of BOS, recognised in specialist studies, such as corneal pigmentation, entropion and distichiasis, were not listed as common conditions in their study of primary care EPRs [12]. Our study of VetCompass Australia EPRs from primary care visits identified common components of Pug ocular disease, including corneal pigmentation (3.6%), ulcerative keratitis (5.5%) and other components, including KCS (3.3%), blepharospasm (2.4%), corneal scarring (1.6%), entropion and neovascularisation (1.1% each) (Table 2).

The sensitivity of the detection of corneal abnormalities was influenced by the primary care context, in which the description of clinical findings was often limited, and we relied on our interpretation of clinical data extracted from the EPRs and note the limited use of additional diagnostic tests. When pugs underwent corneal examinations with specialist techniques [13,39], abnormalities were identified from as early as 16 weeks in most dogs, including corneal pigmentation (usually mild), in 82.4% of pugs [18] and 94.5% of pug eyes [39]. Corneal pigmentation is categorised from very mild (affects less than 2 mm of the limbus), mild (less than 20% of the surface) and moderate (20–50%) to severe (more than 50%), so it appears likely that primary care veterinarians considered it less relevant clinically, and infrequently recorded very mild and mild pigmentation. Corneal vascularisation was reported in 57.9% of eyes and was associated with corneal pigmentation [39] during the specialist examination. Additional ocular abnormalities were identified in more than 70% of pug eyes during the specialist examination, including persistent pupillary membranes, iris hypoplasia and distichiasis [13,39], but were less frequently reported in the UK and Australian Vetcompass studies of primary care EPRs. Considered together, the findings of the many studies on pug BOS suggest insufficient recognition, reporting and active management of eye abnormalities due to the lack of recognition or acceptance of these among owners of, breeders of and veterinarians for the breed. The problems do not appear to be a result of unregulated backyard breeding, as is often claimed, but are widespread in the breed, and across the world. Indeed, corneal pigmentation was more common in registered (87.6%) compared to non-registered pugs (78.8%) in the USA, and among owners of registered pugs in the USA, many did not recognise mild to moderate corneal pigmentation in their dogs [18].

Corneas exposed to environmental factors (e.g., greater cumulative lifetime exposure to solar irradiation, heat and dry conditions in Australia) may have contributed to the more frequent diagnosis of corneal disease compared to the UK [40]. There may also have been differences in breed standards or their application between the two countries. Although the pug breed standards of the ANKC [34] consider the position and morphology of the eyes stating “Never protruding, exaggerated or showing white. Free from obvious eye problems”, no parameters for defining abnormalities of the palpebral fissure or corneal surface have been provided as a guideline for breeders. In particular, the standards do not specify that the eyelids must close fully at rest. Purebred dogs, particularly the brachycephalic breeds, were reported to have a higher prevalence of ophthalmological disorders in the initial UK VetCompass report [41], which sampled the database differently. In a USA specialist study, registered and non-registered pugs both had a very high prevalence of corneal disease [42].

Ulcerative keratitis (5.5%) was the most prevalent ophthalmological condition found in this study, which is consistent with the findings in all breeds [43,44]. Veterinarians did not often record clinical findings that allowed the researchers to distinguish between superficial and the more severe deep and melting ulcers. The association between skull conformation of brachycephalic breeds and corneal disorders is recognised [1,2,3,10], and these breeds were found to be 20 times more likely to suffer from corneal ulceration than non-brachycephalic breeds [2]. Brachycephalic breeds, particularly pugs, were found to have a large palpebral fissure and most were found to be affected by corneal ulcers based on fluorescein staining [10]. A large palpebral fissure is frequently accompanied by shallow orbits, causing exophthalmia, which increases the risk of trauma and subsequent ulceration based on the severity of the protrusion [1,3,10]. Craniofacial abnormalities and abnormal palpebral fissures can be scored but were rarely reported in primary care visits. The mean relative palpebral fissure width was found to be the highest in brachycephalic breeds, including pugs, and dogs with a higher relative palpebral fissure width were found to have a 1.12 times higher risk of developing corneal ulcers [10]. This suggests the measurement of palpebral fissures could be useful in predicting the risk of corneal ulceration in pugs. The protuberance of the globes increases the risk of lagophthalmos, which in turn compromises the distribution of the protective preocular tear film and increases the risk of infections, corneal ulcers and eye prolapse [1,10]. However, only 0.9% of pugs were reported to have exophthalmos, and the length of palpebral fissures was not mentioned in the EPRs for any dogs in this study. The low prevalence of exophthalmos could be due to underreporting because of veterinarians’ perceptions of this conformation as being “normal” for the breed. Investigations of veterinarians’ perceptions of the traits of brachycephalic breeds and the normalisation of dysmorphic features of the breed may be informative.

The high prevalence of corneal ulcers may be a result of reduced corneal sensitivity in brachycephalic dogs [44,45,46,47]. Corneal sensitivity refers to the minimum stimulation on the cornea required to elicit a blink reflex [45]. A lower corneal sensitivity means brachycephalic dogs are less responsive to stimuli that may cause corneal damage, and any corneal lesions in the early stages will be less apparent to dog owners due to the lack of overt signs of ocular pain and discomfort (epiphora, blepharospasm and a raised third eyelid) [10,17]. A study using data from a specialist eye clinic in the UK reported that pugs were commonly diagnosed with a more severe grade of ulcerative keratitis [43]. Additionally, the prevalence of corneal ulceration was found to increase with age based on this study. In light of this, veterinarians could include the fluorescein test as part of the routine physical examinations in pugs with any signs of irritation to check for corneal ulcers. This may ensure early stages of corneal lesions are identified and managed and hence prevent the progression to a stage in which the damage becomes irreversible.

Only 1.1% and 0.3% of pugs were reported to have entropion and distichiasis, respectively, in the current study, which is relatively low when compared to other studies [13,26]. The excessive skin folds in these breeds, particularly the nasal folds near the eyes, in combination with exophthalmos, also predispose them to trichiasis and thus corneal damage [1,2,10]. Entropion and distichiasis have a heritable basis and have been reported in a number of brachycephalic breeds, including pugs and Shih Tzus [48]. Dogs with excessive nasal folds were found to be five times more likely to suffer from corneal ulcers [10]. Measurements of over-nose wrinkle and nasal fold width have been used in another study to investigate the effect of nasal folds on corneal disorders [10,19]. However, there were no cases of trichiasis reported in pugs in the year of study, and no records were made regarding nasal folds in the examination notes of the EPRs analysed in this study. Chronic irritation from the common eyelid conditions in pugs, such as distichiasis and entropion, has been reported to contribute to keratopathies [10,13,19,49,50]. In one study, 94.1% of pugs in a show had entropion while 11.9% of them had distichiasis [19]. This confirms the under-recognition of these defects, which were not identified at the pre-show health check, and the dogs were not disqualified from showing or breeding. It was found that the severity of pigmentary keratitis was positively associated with the severity grade of the medial entropion of the lower eyelid [16]. However, no EPR recorded the grading of the entropion, so the low level of reporting likely reflects the under-recognition of these dysmorphias as abnormalities, which might result in a missed diagnosis and insufficient surgical treatment. Nonetheless, the association between entropion, excessive nasal folds, distichiasis and the risk of corneal pathologies found in other studies demonstrated the significance of explicit examinations, the recognition of subtle abnormalities, and the measurement and recording of these conditions during routine clinical examinations [10]. Pugs may show fewer signs of eye irritation, or these may be considered normal behaviour by their owners (e.g., “he has always rubbed his eyes a lot since he was a puppy”), leading to the under-presentation and underdiagnosis of these issues. Early identification of structural issues with gradings could allow for timely interventions with surgery to correct the anomalies and hence prevent the progressive development of corneal conditions. While the breed club recommends ophthalmological examinations when issues are identified, the systematic measurement of the palpebral fissure, the scoring of craniofacial deformities, and the assessment of nasal folds and the medial entropion of the upper and lower eyelids could be incorporated into the breed standards and pre-show examinations. The recognition of the high frequency of abnormalities would help to correct the misperception of these as “normal” morphologies in this breed and increase focus on their elimination from breeding animals.

Corneal pigmentation (3.6%) was the second most prevalent ophthalmological disorder in this study and was also found to be prevalent in pugs presented to ophthalmology units of the Veterinary Schools in Vienna, Cornell, Las Palmas, Lisbon and Leipzig [13,39,42,49]. The pugs with corneal pigmentation were older, with a median of 7.6 years, in agreement with a current report stating that age and past ocular disease are risk factors [39]. In pugs with corneal pigmentation (*n* = 48), 18.8% of them had concurrent corneal ulcers (*n* = 9), 39.6% had concurrent KCS (*n* = 19) and 10.4% had concurrent keratitis (*n* = 5). Several studies have investigated the association between KCS, corneal pigmentation and corneal ulcers in dogs. Corneal disorders consisted of a range of presentations including inflammation, pigmentation, ulceration, scarring, lipidosis and other keratopathies, many of which are non-specific biological responses to various irritating stimuli, such as trauma, mechanical abrasions, tear-film deficiencies including KCS and immune-mediated keratitis [8,15,18]. A significant association was found between KCS and the presence of corneal pigmentation in a study in Vienna [13]. A number of dogs with corneal pigmentation were found to have concurrent corneal ulcers and KCS, which reflects that the cornea of pugs is prone to external trauma and irritation due to their protuberant globes and the likelihood of chronic injury.

KCS (3.3%) was the third most prevalent ophthalmological abnormality found, but quantitative and qualitative KCS were not differentiated in this study. There are two types of KCS, quantitative and qualitative, and each is a result of the deficiency of a different component of the tear film. Qualitative KCS is a result of the deficiency of the aqueous, or the middle, layer of the tear film, which can be assessed via the Schirmer tear test (STT) [13,15,17]. Quantitative KCS is a result of deficiencies in both the mucin, the inner layer, and lipid, the outer layer, of the tear film which can be identified by the tear break up time (TFBUT), with the use of topical fluorescein to define the areas of mucin absence [13,15,17] or a Meibometer to measure the level of lipids on the eyelid margin [13,22,23]. Tear quality can be evaluated with the tear ferning test (TFT) and has been shown to be abnormal in pugs with pigmentary keratitis [39]. Most of the KCS diagnosed in this study were based only on the result of STT. Based on the EPRs, no patients were offered TFBUT or TFT or were offered referral to ophthalmologists to further investigate the cause of the epiphora when the STT result was normal. Since data were collected from the primary care veterinary practice records in Australia, most of the clinics might have only been equipped with basic ophthalmological diagnostic tools but not more advanced tools such as a meibometer. Without identification by qualitative assessment, the actual prevalence of KCS is likely to be underreported. This warrants a more thorough approach in diagnosing and managing pugs that are showing signs of KCS, such as excessive eye discharge and blepharospasm. Artificial tears would be a treatment option when there are signs of KCS to maintain the protective tear film barrier of the eye. If no improvement was observed over a few weeks, a further investigation using TFBUT or referral should be considered to prevent the development of secondary corneal disorders. Among the pugs with corneal ulcers (*n* = 73), only 5.5% of them were reported to have concurrent KCS (*n* = 4). The prevalence of KCS was found to be relatively low in pugs compared to other brachycephalic breeds in a referral population [42]. Another study found a marked number of pugs had corneal ulceration in the absence of KCS [13]. This suggests that external trauma and exposure might be a more common cause of corneal ulcers than KCS, although it may also be a contributing factor. There is a complex association between KCS, corneal pigmentation and corneal ulcers [12,49]. These conditions are responses to chronic irritation and inflammatory changes which affect the protective barrier functions of the cornea and its tear film. Further studies using 10-year VCA data would be useful to identify the developmental course of clinical corneal conditions in pugs and provide guidance to veterinarians on monitoring the progression of ophthalmological disorders.

Corneal disorders were associated only with age, among the demographic factors investigated. The most prevalent ophthalmological conditions in senior dogs (7+ years) were corneal pigmentation (median age: 7.6 years), KCS (10 years) and keratitis (7.7 years). Quintana et al. also state that the risk of pigmentary keratitis increases significantly in pugs with a median age of 8 years or older, compared to younger age groups [39]. The association of age with corneal disorders was not surprising since there are age-related changes in components of the ocular surface [21,25]. These changes include decreased lacrimal gland secretion and the altered composition of secretions [25], as well as alterations in the meibomian gland and the meibum lipids [51]. Besides changes in the ocular surface, the density of keratocyte and nerve terminals, as well as decreased numbers of endothelial cells with age [52,53], diminish the capacity and rate of repair of corneas in older dogs. These ocular changes may contribute to the higher prevalence of corneal pigmentation and keratitis in senior dogs. The early detection of corneal pigmentation could allow for early interventions and prevent the development of other corneal conditions since corneal pigmentation was reported in younger pugs than KCS and associations have been found between corneal pigmentation, KCS and corneal ulcers [13]. A higher prevalence of neovascularisation and exophthalmos were found in pugs with a median age of 1 year in the current study. Neovascularisation is a normal ocular response to infection, trauma and intraocular events such as glaucoma, uveitis and phthisis bulbi [15]. The young median age of dogs with neovascularisation (median age: 1.0 year) suggests that ophthalmological conditions and corneal damage have commenced at an early age and should prompt thorough routine ophthalmological examinations to identify other abnormalities early in the disease process. Exophthalmos is a congenital ophthalmological condition that is related to skull conformation in pugs [1,2,3,10], can occur subsequent to trauma and other conditions and was most frequently noted in the EPRs for younger dogs.

### 4.2. Demographics

Pugs were more often neutered in Australia compared to the UK [8]. The pugs in this study were young with a median age of 3 years, as in the UK, with rising registrations in the past two decades in both countries [8,54]. Since some of the corneal problems, such as corneal pigmentation and scarring, are responses to chronic irritation, their prevalence may underrepresent the rates that would be seen in a more mature population. The prevalence of overweight/obese pugs in the current study was 20.2% (*n* = 266, 95% CI: 18.1–22.4), which is high compared to 7.1% of dogs across all breeds [55] and 13.2% of pugs in the UK [8] using a similar methodology. Multiple studies have shown that pugs have a higher propensity to become overweight/obese than other breeds [8,41,56], which may be related to the high frequency of BOAS and myelopathy. The most common ways to evaluate overweight dogs (those exceeding 15% of their ideal weight) are the BCS and bodyweight [57]. The pug breed standards of the ANKC [34] state the ideal bodyweight is between 6.3 and 8.1 kg. Hence, pugs with a bodyweight above 9.3 kg are at risk of overweight. Yet, a small percentage of dogs above 12 months old with a bodyweight over 9.3 kg were not reported as overweight. This may be because these dogs had a larger frame, or a higher level of bodyfat coverage was perceived to be a typical trait of the breed. The phrases used to describe the general appearance of pugs in the breed standards are “decidedly square and cobby” [34]. Based on this description, increased fat coverage and loss of defined body contours such as the waist might have been normalised among the owners and veterinarians, contributing to the large amount of missing BCS data. There is evidence of insufficient recognition of obesity in the breed, as the average BCS of pugs attending a dog show in the Netherlands was six out of nine, which was the second highest average BCS among all breeds evaluated [57]. No association was found between obesity and corneal conditions; however, obesity is a clinically significant disorder in dogs, particularly in pugs because it increases the severity of other conditions, including BOAS, heat and exercise intolerance, cardiovascular and orthopaedic diseases, diabetes mellitus and anaesthetic risks [56,57,58]. The prevalence of overweight pugs in the young population in this study is concerning; however, BCS was infrequently recorded. Considering the associated comorbidities and reversible nature of the disorder, addressing overweight/obesity should be a health priority in pug veterinary visits.

### 4.3. Limitations of the Study

The limitations of this study relate to the use of primary care veterinary records that were not created for the purpose of research, so they may not describe all findings or report on all the abnormalities observed, and the lack of use of more advanced ophthalmological diagnostic tests, both factors which contribute to the under-recognition and underreporting of BOS abnormalities. In this study, all clinical records were examined in detail in order to detect terms and descriptions used by clinicians to describe ocular disease. The lack of availability of ophthalmological diagnostic equipment (other than an ophthalmoscope) and lack of familiarity with tests such as the TFBUT in primary veterinary care practices reduce the likelihood that certain ophthalmological conditions will be diagnosed (e.g., qualitative KCS) and also reduce the stringency of ophthalmological conditions that are described, which increases type II errors.

Obesity and overweight conditions were substantial problems in this study, as in other studies of pugs [8]. They may also have been under-recognised because the BCS was only reported for 29% of the study population, with veterinarians mainly recording the BCS when it was abnormal. The BCS of dogs with a weight 15% above the breed standard should be routinely recorded. BCS is a semi-quantitative scoring system that measures the morphology of dogs based on the palpation of fat distribution [59]. However, the non-numeric evaluation of BCS is subjective. For an individual adult dog, bodyweight provides a more objective numeric measure and is more sensitive to detecting subtle weight changes for an individual animal. Nevertheless, it is subject to large variations within a breed based on sex, exercise level and stature and differs between individuals [58]; hence, it was not considered when determining if a patient is overweight in this study. As pugs are at increased risk of obesity, weight and BCS should be recorded at each veterinary visit.

Minor issues with VetCompass Australia data were the inability to ensure that all the dogs that attended more than one veterinary business were identified and their records were combined, as well as the lack of a neutering date for the dogs that were previously neutered.

The results of this study provide a framework to guide ocular health priorities for primary care veterinary treatment of pugs. A more rigorous ophthalmological examination with an ophthalmoscope should be considered as part of the wellness exam in this breed. Examinations should include the use of more advanced diagnostic tests, starting from puppy visits and continuing through the pug’s life to allow for the identification of ophthalmological pathologies at their onset and to commence early interventions. The greater use of tests of corneal function and grading of the severity of findings could improve the recognition and monitoring of the progression of ophthalmological abnormalities such as entropion, corneal pigmentation and corneal ulcers. This would ensure that low-grade cases are detected and receive timely intervention. Veterinarians could better educate dog owners to recognise the risks associated with brachycephalic conformational features, such as a large palpebral fissure, the failure to close the eyelid, distichiasis and excessive nasal folds, to help owners understand that these are abnormalities which predispose dogs to corneal disorders and to energise owners to identify problems and seek treatment early. The reformation of pug breed standards to address palpebral fissure width, eyelid closure, muzzle length and nasal folds, along with the early removal of dogs with signs of corneal irritation, pigmentation and ulceration from showing and breeding, are necessary to improve the health and welfare of the breed.

## 5. Conclusions

The prevalence of ophthalmological conditions and corneal disorders in pugs in Australia was high compared to similar studies of pugs and other breeds in the UK. Corneal ulcers, corneal pigmentation and KCS were the most prevalent ophthalmological pathologies, with many other BOS-related conditions identified. Age increased the risk of corneal disease. Progressive corneal conditions (corneal pigmentation, KCS and non-ulcerative keratitis) were more often diagnosed in older dogs; however, entropion and ulcerative keratitis were identified in younger dogs. Many dogs had more than one ocular condition; most conditions were likely to be persistent and/or recurrent and to reduce patient well-being. There is an opportunity to reduce the suffering associated with ocular conditions in pugs through the greater recognition of the breed’s risk, early identification and effective treatment, along with strengthened breed standards for ocular conformation.

## Figures and Tables

**Table 1:** Demographics of pugs and pugs with ophthalmological disorders attending primary veterinary practices in Australia in 2017 (*n* = 1318).

Variable	Category	Percentage (%) of the Total Population	Percentage of Dogs with Ophthalmological Disorder(s) in Each Category (%)
Sex	Male	53.9	11.7
	Female	46.1	17.4
Neuter status	Entire	26.3	11.6
	Neutered	73.7	15.3
Age	Puppy (0–12 months)	23.3	7.5
	Adult (1–6 years old)	50.2	11.8
	Senior (7+ years old)	26.5	25.2
Body condition	Not overweight	8.4	21.6
	Overweight	17.1	17.7
	Obese	3.0	22.5
	Not reported	71.4	12.3
Coat colour	Fawn	60.4	14.8
	Black	26.3	13
	Brown	9.7	15.6
	Apricot	1.3	5.9
	Other	0.8	20
	Silver	0.5	28.6
	Brindle	0.3	0
	No reported	0.8	10

**Table 2:** Prevalence of the 10 most common ophthalmological disorders recorded in pugs attending primary care veterinary clinics in Australia in 2017 (*n* = 1318).

Specific Ocular Conditions	Median Age (Years)	Prevalence %	95% CI
Ulcerative keratitis	2.0	5.5	4.4–6.9
Corneal pigmentation (melanosis or unspecified)	7.6	3.6	2.8–4.8
Keratoconjunctivitis sicca (KCS)/dry eye	10.0	3.3	2.5–4.5
Blepharospasm	2.8	2.4	1.7–3.4
Keratitis (pannus, pigmentary or unspecified)	7.7	2.1	1.5–3.1
Conjunctivitis (infectious, non-infectious or unspecified)	4.0	1.7	1.2–2.6
Corneal scar	2.0	1.6	1.0–2.4
Entropion	3.0	1.1	0.7–1.9
Neovascularisation	1.0	1.1	0.7–1.9
Nuclear sclerosis	10.0	1.1	0.7–1.9
Epiphora	2.0	1.1	0.6–1.8
Exophthalmos (including proptosed eyes)	1.0	0.9	0.5–1.6
Blindness (unilateral, bilateral or unspecified)	11.2	0.6	0.3–1.2

**Table 3:** Specific ophthalmological disorder findings in orbit, eyelids, tear and nasolacrimal systems and cornea in affected pugs attending primary care veterinary practices in Australia in 2017.

Ocular Anatomical Groups	Specific Ocular Conditions	Prevalence %
Cornea (*n* = 176) Prevalence = 13.4%	Ulcerative keratitis (descemetocele, melting, infective or unspecified)	5.5
	Corneal pigmentation (melanosis or unspecified)	3.6
	Keratitis (pannus, pigmentary or unspecified)	2.1
	Corneal scarring	1.6
	Keratopathy	0.3
	Corneal lipidosis	0.08
	Keratoconjunctivitis (unspecified)	0.08
Eyelids (*n* = 58) Prevalence = 4.4%	Blepharospasm	2.4
	Entropion	1.1
	Distichiasis	0.3
	Eyelid mass	0.2
	Blepharoedema	0.15
	Chalazion (meibomian cyst)	0.08
	Meibomian epithelioma	0.08
	Meibomian adenoma	0.08
Tear and nasolacrimal systems (*n* = 57) Prevalence = 4.3%	Keratoconjunctivitis sicca/dry eye	3.3
	Epiphora	1.1
Orbit and other (*n* = 14) Prevalence = 1.1%	Exophthalmos	0.76
	Proptosed eye/s	0.15
	Microphthalmia	0.08
	Glaucoma	0.08

**Table 4:** Univariate logistic regression results for risk factors associated with corneal disorders in 1318 pugs attending primary care veterinary practices in Australia in 2017 (*p* < 0.05).

Variable	Category	Odds Ratio	95% CI	*p*-Value
Sex	Female	Reference		
	Male	1.05	0.75–1.46	0.782
Neuter status	Neutered	Reference		
	Entire	1.42	0.96–2.14	0.0872
Age (Categories)	Puppy	Reference		
	Adult	1.79	1.09–3.06	
	Senior	3.68	2.22–6.35	<0.0001
Age (Continuous)		1.11	1.07–1.15	<0.0001
Body condition	Not overweight	Reference		
	Overweight including obesity	1.01	0.57–1.86	0.967
Coat colour	Black	Reference		
	Brown	1.22	0.66–2.18	
	Fawn	1.06	0.72–1.57	
	Other (apricot, brindle, silver and other incorporated)	1.13	0.37–2.82	0.9327

**Table 5:** Multivariate logistic regression results for risk factors associated with corneal disorders in 1318 pugs attending primary care veterinary practices in Australia in 2017 (*p* < 0.05).

Variable	Category	Odds Ratio	95% CI	*p*-Value
Age	Puppy	Reference		
	Adult	1.76	1.06–3.06	
	Senior	3.61	2.13–6.37	<0.0001
Neuter status	Neutered	Reference		
	Entire	1.05	0.70–1.62	0.8131

## Data Availability

The datasets are not publicly available, but were supplied by VetCompass Australia, to whom enquiries should be directed.
